# The electrical properties of isolated microtubules

**DOI:** 10.1038/s41598-023-36801-1

**Published:** 2023-06-22

**Authors:** Brenda C. Gutierrez, Horacio F. Cantiello, María del Rocío Cantero

**Affiliations:** Laboratorio de Canales Iónicos, Instituto Multidisciplinario de Salud, Tecnología y Desarrollo (IMSaTeD, CONICET-UNSE), Santiago del Estero, Argentina

**Keywords:** Nanoscale biophysics, Single-molecule biophysics

## Abstract

This study examines the electrical properties of isolated brain microtubules (MTs), which are long hollow cylinders assembled from αβ-tubulin dimers that form cytoskeletal structures engaged in several functions. MTs are implicated in sensory functions in cilia and flagella and cellular activities that range from cell motility, vesicular traffic, and neuronal processes to cell division in the centrosomes and centrioles. We determined the electrical properties of the MTs with the loose patch clamp technique in either the presence or absence of the MT stabilizer Paclitaxel. We observed electrical oscillations at different holding potentials that responded accordingly in amplitude and polarity. At zero mV in symmetrical ionic conditions, a single MT radiated an electrical power of 10^–17^ W. The spectral analysis of the time records disclosed a single fundamental peak at 39 Hz in the Paclitaxel-stabilized MTs. However, a richer oscillatory response and two mean conductances were observed in the non-Paclitaxel MTs. The findings evidence that the brain MTs are electrical oscillators that behave as "ionic-based" transistors to generate, propagate, and amplify electrical signals.

## Introduction

Microtubules (MTs) are long hollow cylinders assembled from αβ-tubulin dimer subunits^1–3^. The tubulin dimers assemble into linear pearl necklace-type formations, the protofilaments, which build into an enclosed polymer, the microtubule. Previous studies by ours and other groups had determined both experimentally and theoretically the ability of MTs to act as nonlinear transmission lines capable of electrical signal amplification^4,5^. Different assemblies of brain MTs, including sheets and bundles, generate strong electrical oscillations^6^, whose behavior is mechanistically consistent with organic electrochemical transistors, where a gate region would drive the synchronous open-close cycles of ion-permeable nanopores that elicit an electrodiffusional circuit, supporting both amplification and self-sustained current- (and voltage-) oscillations^7,8^. From a structural viewpoint, this electrical activity may be linked to the mechanical changes in the ensembles of the MT subunits^9^. MTs display memristive device behavior that may underlie the gating mechanism of the MT conductance^10,11^. The different structural lattices of the MT surface generated by the lateral apposition of protofilaments produce at least two types of nanopores at either interdimer interfaces or intradimer interfaces^12^. Thus, the electrical phenomenon would implicate changes in nanopore conductance^7^. Presently, the electrodynamic properties of isolated MTs remain unknown.

Herein, we explored the ability of single MTs to generate electrical signals by the voltage clamp technique. Isolated MTs generated electrical oscillations at holding potentials different from zero mV. Spontaneous changes in amplitude were observed in response to both the magnitude and polarity of the electrical driving force. The spectral analysis of the time records disclosed fundamental frequencies with prominent peaks between 43 and 47 Hz and around 90 Hz. Interestingly, we observed functional differences in the Paclitaxel-stabilized MTs. Paclitaxel stabilization locked the electric oscillatory behavior into a fundamental frequency of approximately 39 Hz. The electrical oscillatory information produced by MTs may be central to the function of neuron behavior.

## Results

### Electrical recordings of non Paclitaxel-stabilized isolated microtubules

**Figure 1:**
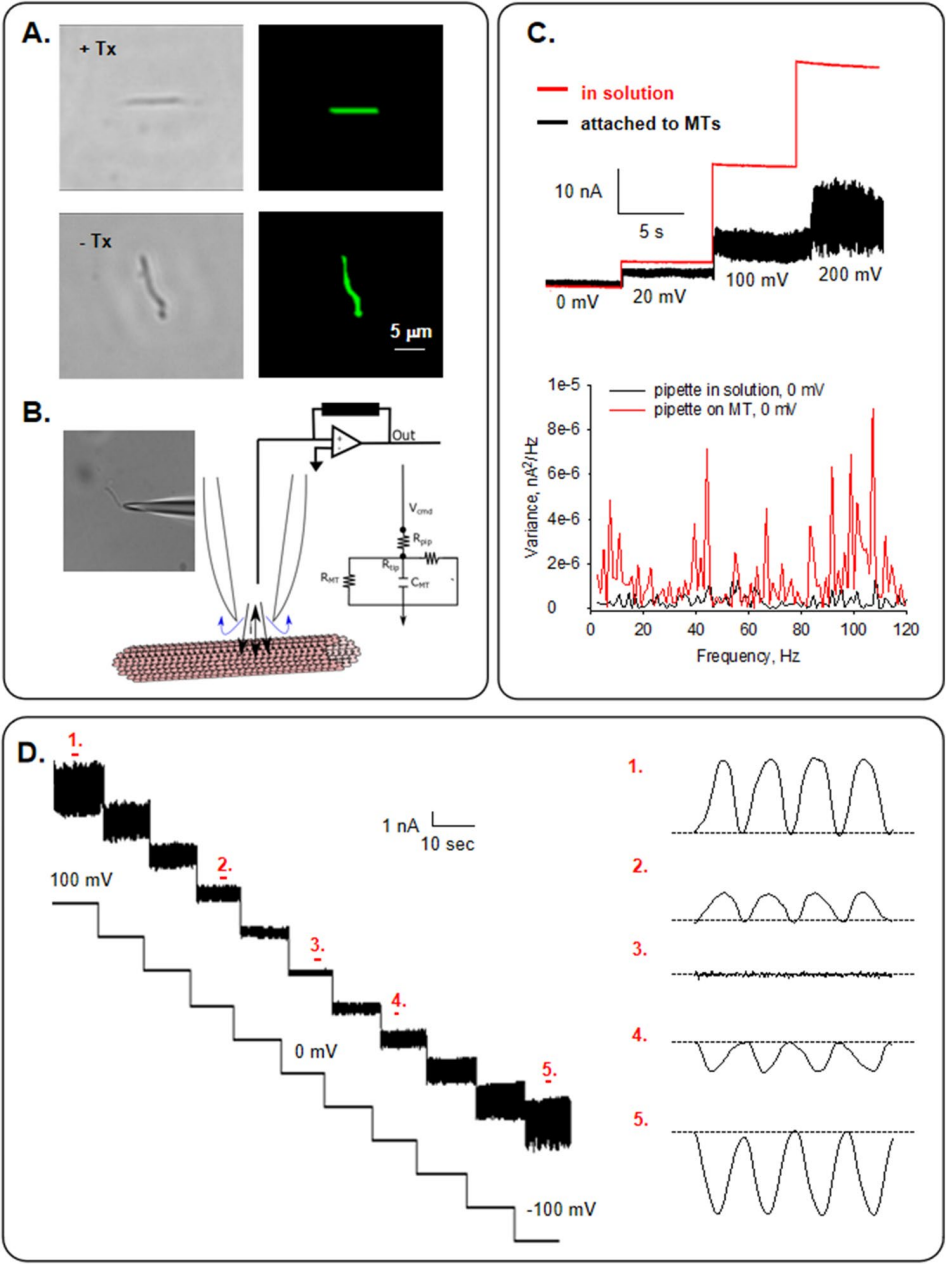
Experimental setup and electrical recordings from isolated brain microtubules. (**A**) DIC (Left) and fluorescent (Right) images of isolated MTs (×60), prepared in the presence (Top) or absence (Bottom) of Paclitaxel. (**B**) Schematics of the loose patch configuration used in the study and an image of a pipette tip holding an MT (Left). (**C**) Top. Currents elicited by different holding potentials obtained with the pipette in solution before (Red) and after (Black) attachment to an isolated MT. Bottom. Frequency spectra at 0 mV for the pipette in solution (Black) and after MT apposition (Red). Please, note that notch filtering was applied to eliminate contaminating line frequencies at 50 and 100 Hz. (**D**) Electrical recordings from an isolated MT at different holding potentials (± 100 mV) as indicated. Expanded tracings on the Right show electrical oscillations of regions numbered “1” through “5”. Applied voltages represented the driving potentials at the patch clamp amplifier.

Commercial bovine brain tubulin was polymerized either in the presence or absence of Paclitaxel (Fig. 1A), as described in “Materials and methods”. We conducted the present study to obtain electrical recordings from isolated MTs. The experiments were conducted under symmetrical conditions with identical saline composition in both patch pipette and bathing solution. Isolated MTs were immunolabeled and visualized under combined DIC and fluorescent microscopy by exposure to both a primary anti-α-tubulin antibody and a FITC-fluorescent secondary antibody (Fig. 1A). We observed that in both conditions, the MTs had sizes comparable with those previously reported^13–15^.

**Figure 2:**
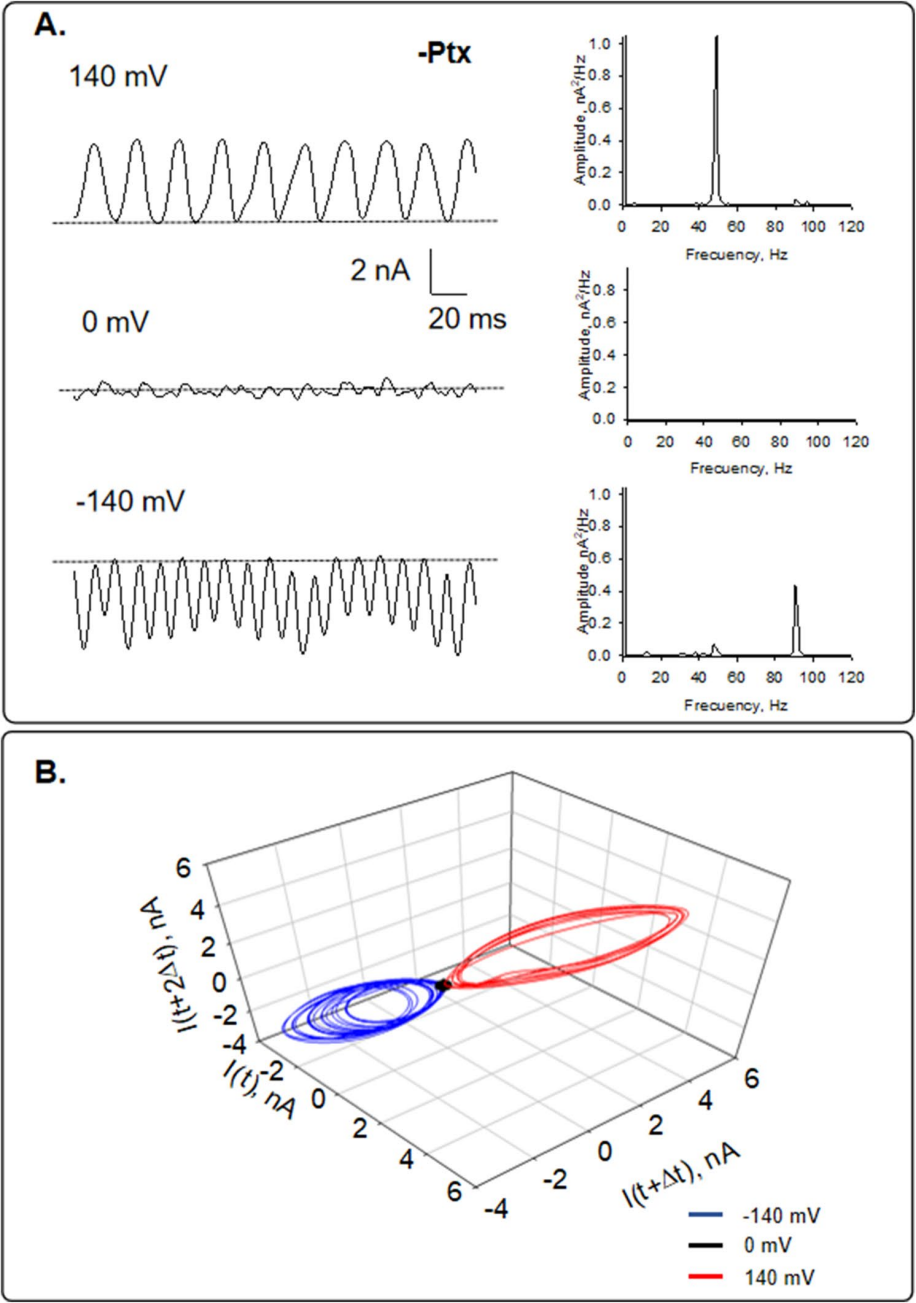
Current oscillations of isolated MTs in the absence of Paclitaxel. (**A**) Left. Tracings of current fluctuations at different holding potentials of an isolated MT in the absence of Paclitaxel (−Ptx). Data are representative of* n* = 5 experiments. Right. Linear–linear plots of the Fourier power spectra obtained from tracings shown on the Left. The fundamental frequencies can be observed on the spectra except for zero mV. (**B**) Three-dimensional phase-space portrait showing limit cycles for different holding potentials (− 140 mV in Blue, 0 mV in Black, and 140 mV in Red). Trajectory at negative holding potentials shows period doubling. The Delay time for the first and second derivatives adopted for the phase portraits was 0.1 ms.

Experiments were conducted with small tipped (4 μm^2^) pipettes. Apposition of the pipette tip onto an isolated MT (Fig. 1B) only increased the seal resistance to 1.24 ± 0.29 MΩ reaching 12.84 ± 5.20 MΩ, *n* = 14, Median 6.50 MΩ, and a range of 2.83 to 63.3 MΩ. The approach rendered a loose patch configuration as previously used with brain MT bundles^8^, in contrast to the high seal resistances usually obtained with MT sheets^7,8^. Isolated MTs formed in the absence of Paclitaxel were initially voltage-clamped at zero mV, showing no electrical activity in any successful experiments (*n* = 14). However, under these conditions, the variance of the current at 0 mV was statistically different from that of the pipette in solution (solution vs. MT-attached: 1.17 ± 0.15 pA^2^, *n* = 6, vs. 2.27 ± 0.49 pA^2^, *n* = 6, p = 0.04) that was concomitant with the presence of oscillatory peak frequencies (Fig. 1C). MTs displayed spontaneous, self-sustained electrical oscillations in 69% of cases (20/29) in direct response to the magnitude and polarity of the electrical stimulus (Figs. 1D and 2A). The most prominent frequency peaks with the simplest oscillations observed were at 43–47 Hz and 90 Hz at negative holding potentials. At positive holding potential, only 43–47 Hz was observed, at least at the beginning of the experiments. These oscillations showed monoperiodic limit cycles, as shown in the three-dimensional phase portrait (Fig. 2B). At zero mV, only the 50 Hz line interference frequency was present.

### Electrical recordings of Paclitaxel-stabilized isolated microtubules

**Figure 3:**
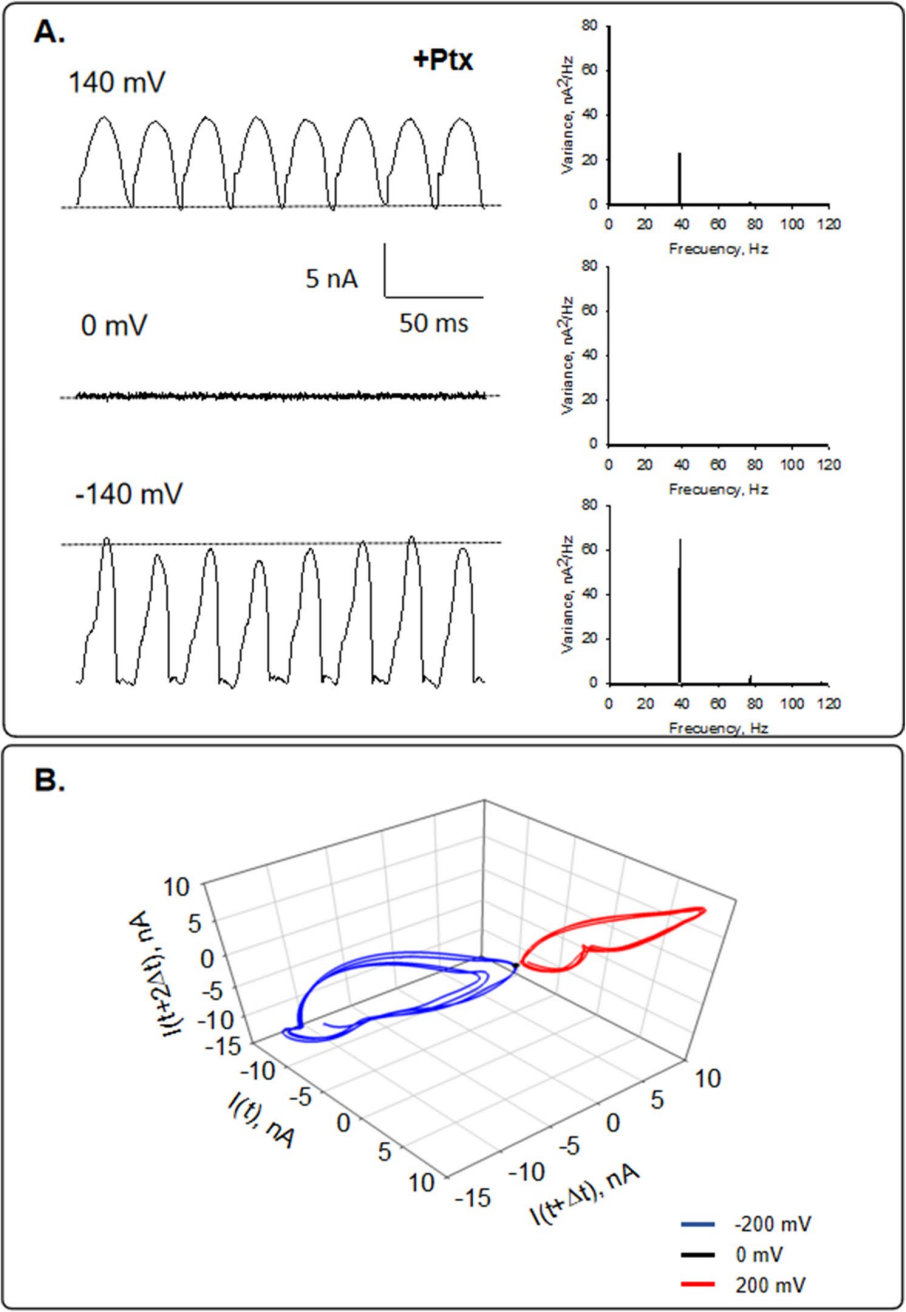
Current oscillations of Paclitaxel-stabilized isolated MTs. (**A**) Left. Current fluctuations of an isolated MT in the presence of Paclitaxel (+Ptx) at different holding potentials. Data are representative of *n *= 3 experiments. Right. Linear–linear plots of the Fourier power spectra obtained from tracings shown on the Left. The fundamental frequencies can be observed on the spectra. (**B**) Three-dimensional phase-space portrait showing limit cycles for different holding potentials (− 200 mV in Blue, 0 mV in Black, and 200 mV in Red). Trajectory at negative holding potentials shows period doubling. The Delay time for the first and second derivatives adopted for the phase portraits was 0.1 ms.

To evaluate the potential effect of Paclitaxel stabilization on the oscillatory behavior, isolated MTs obtained with the Paclitaxel protocol were also voltage-clamped (*n* = 3). At positive and negative holding potentials (± 200 mV), MTs also displayed spontaneous, self-sustained electrical oscillations (Fig. 3) in direct response to the magnitude and polarity of the electrical stimulus. However, the power spectrum revealed a prominent frequency peak at 39 Hz, similar to MTs sheets and bundles^8,16,17^, and a small peak near 74 Hz, not previously observed in any other preparation. This suggests that the assembly of the MTs has a relevant effect on the frequencies at which MTs oscillate. These oscillations showed monoperiodic limit cycles, as is shown in the three-dimensional phase portrait (Fig. 3B). At zero mV, only the 50 Hz power line interference frequency was observed.

### Current-to-voltage relationship and frequencies of non-Paclitaxel stabilized isolated microtubules

**Figure 4:**
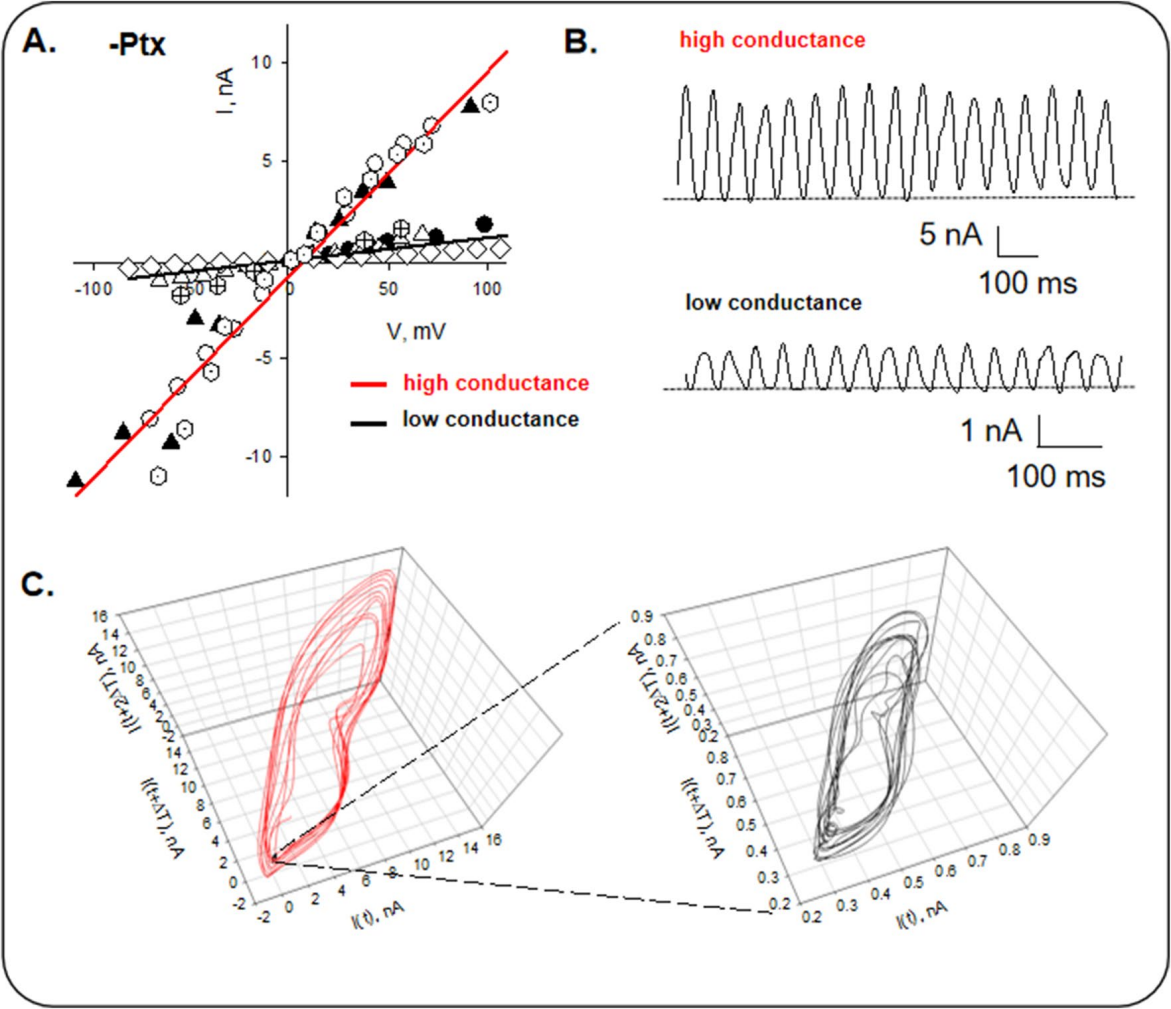
Current-to-voltage relationships of non-Paclitaxel stabilized isolated MTs. (**A**) Mean current-to-voltage relationships were obtained for mean currents at different holding potentials. Different symbols represent individual experiments. Two high and low conductance levels are present (*n* = 3 and 4, respectively). Voltages are corrected by pipette and seal resistance. (**B**) Examples of current oscillations at 100 mV (holding voltage) are shown. (**C**) Three-dimensional phase-space portraits show monoperiodic limit cycles for both conductance values. Please note that except for their magnitude, the trajectories are identical. The Delay time for the first and second derivatives adopted for phase portraits was 0.1 ms.

The mean oscillatory currents were linear concerning the corrected holding potential (Fig. 4A,B), showing at least two different "high" and "low" conductances of 103.0 ± 3.5 nS (*n* = 3) and 11.3 ± 1.2 nS (*n* = 4), respectively. Freedman et al.^12^ calculated single MT current–voltage relationships from a cation conductance circuit model with a conductance in the order of 10 nS. Three-dimensional phase-space portraits showing monoperiodic limit cycles were observed for both conductance levels (Fig. 4C).

**Figure 5:**
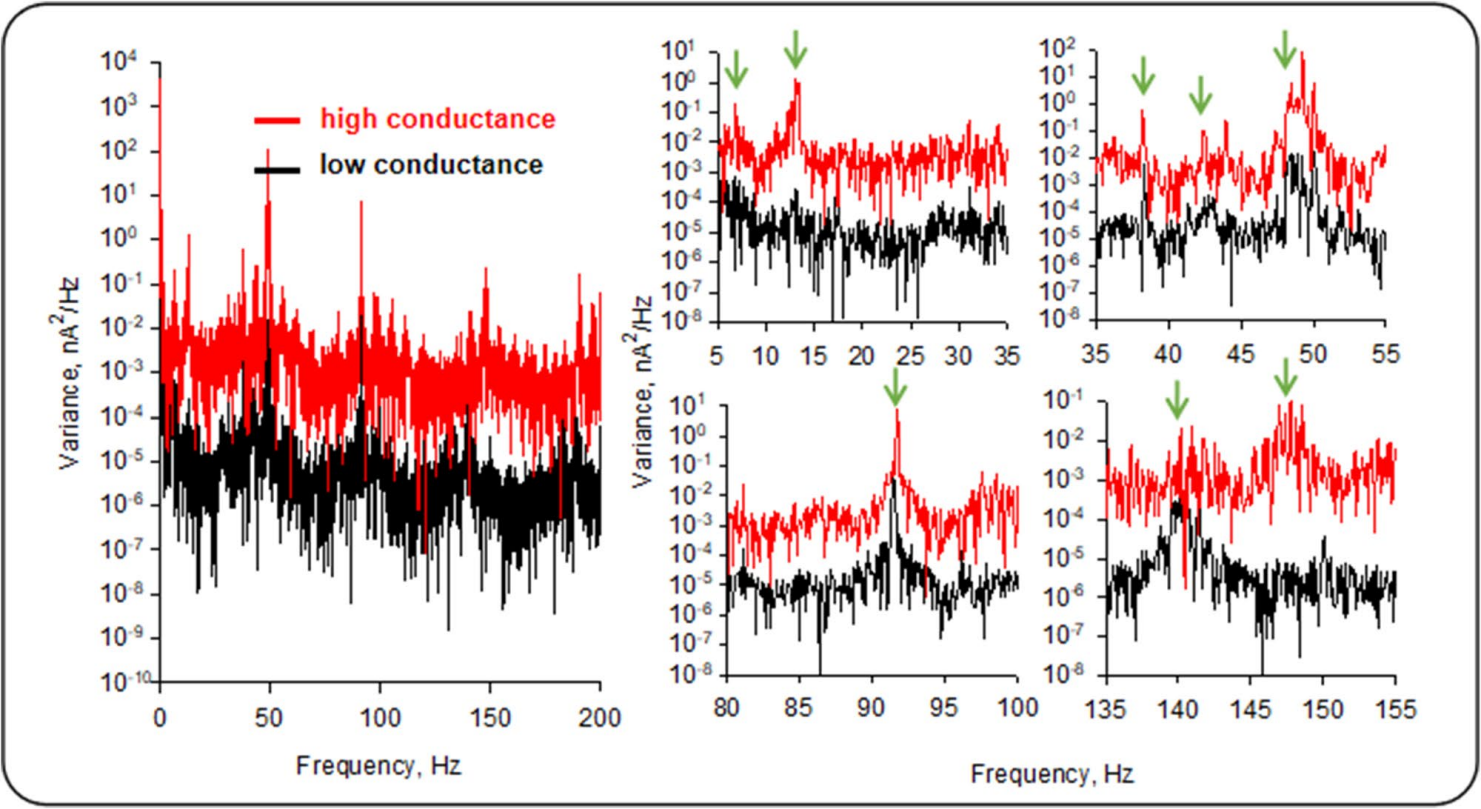
Oscillatory behavior of the high and low conductance MT regimes. Left. The high (Red) and low (Black) conductance power spectra are shown. Right. Expansions of power spectra on the Left to show in detail the frequency domains. Peaks at 13 Hz and 147 Hz are not present in the low conductance state. Green arrows indicate specific frequency peaks.

Fundamental frequencies were seen at approximately 7, 13, 28, 39, 47, 90, 140, and 147 Hz (Fig. 5). However, not all the frequencies were prominent in experiments with low and high conductance. The 13 and 147 Hz were observed only in the high conductance experiments, while the 140 Hz was noticeable in the low conductance experiments.

**Figure 6:**
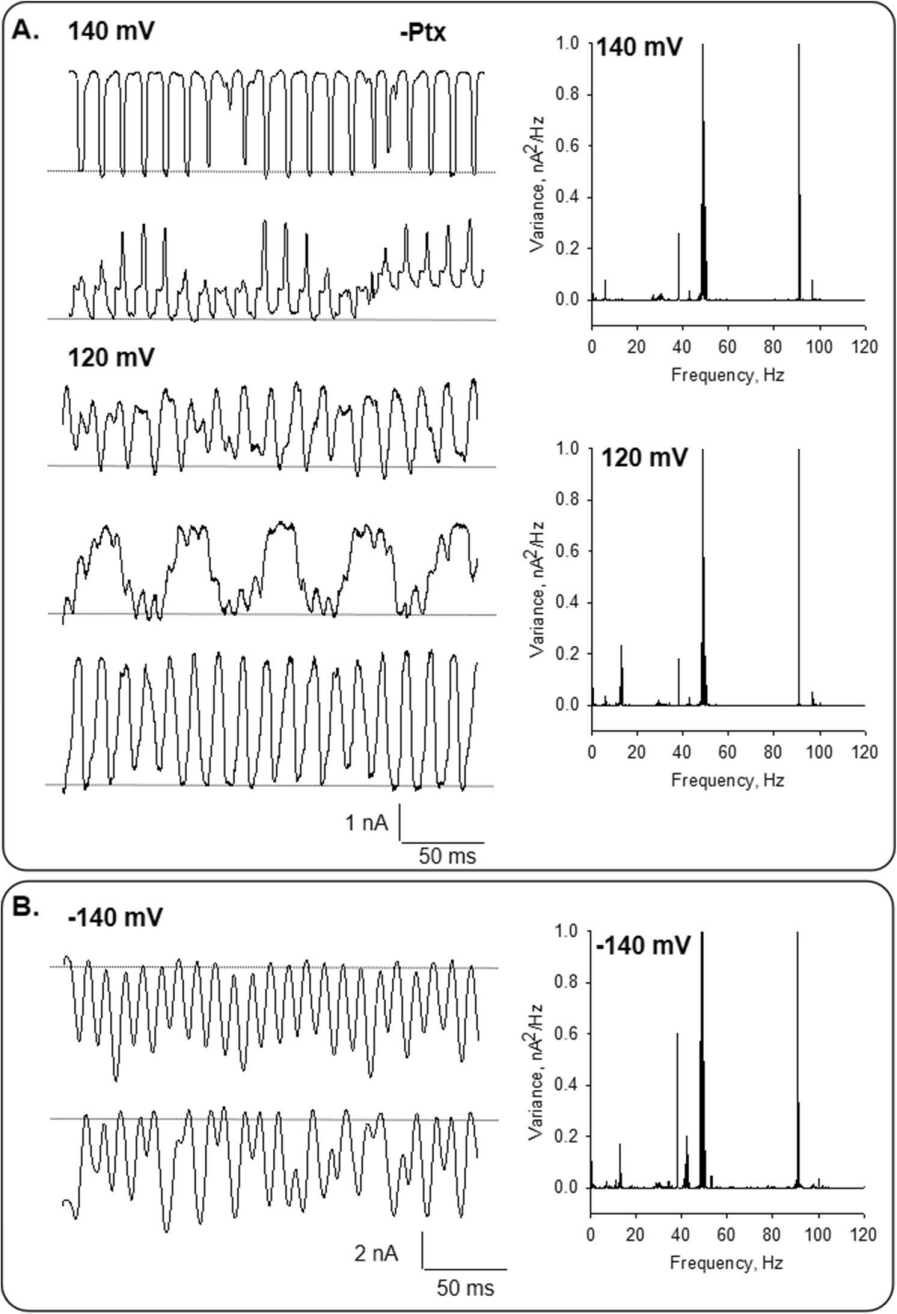
Effect of holding potential on the current oscillations of brain MTs. (**A**) Left. Example of changes observed in the oscillatory behavior of an MT at 140 mV and 120 mV. Tracings represent different instances of the same record. Right. The panel shows the power spectrum from oscillatory currents shown in tracings on the Left. (**B**). Left. Example of changes observed in the oscillatory behavior of an MT at − 140 mV. Tracings represent different instances of the same record. Right. The panel shows the power spectrum from oscillatory currents shown in tracings on the Left.

A richer oscillatory behavior was observed when the same holding potential was applied for several minutes (Fig. 6). In these instances, the oscillation patterns observed were variable in the time with low frequencies periods alternating with high frequencies periods, making evident a more coherent activity at different fundamental frequencies (Fig. 6) for positive and negative potentials, respectively.

### Comparison between MT sheets and non-Paclitaxel stabilized isolated MTs

**Figure 7:**
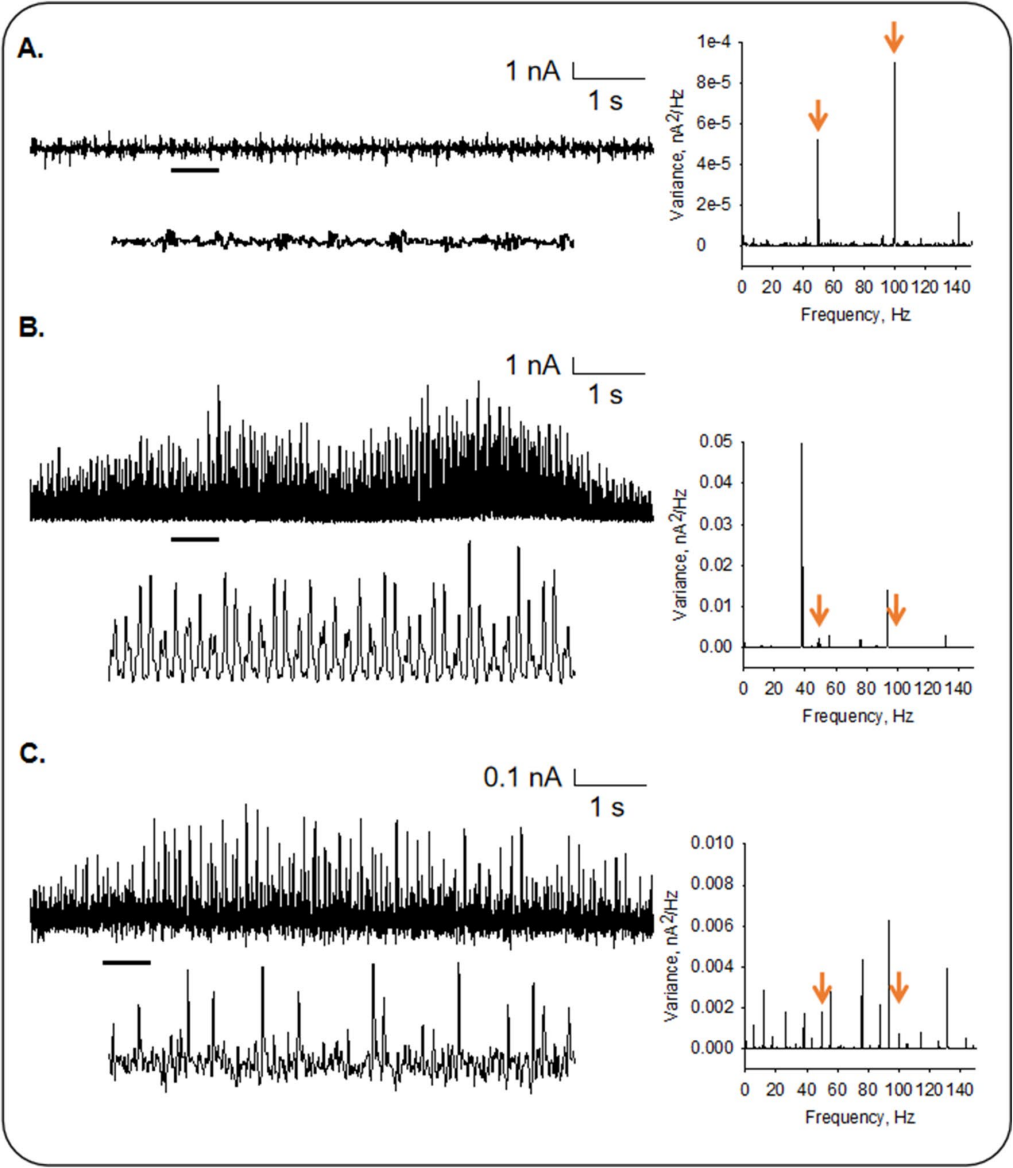
Differences in oscillatory behavior between isolated MTs and MT sheets. Recordings from the pipette in solution (**A**), a voltage-clamped MT sheet (**B**), and an isolated MT (**C**). Representative experiments of *n* = 5, 5, and 4, respectively. Data were taken at 20 mV. Expanded tracings are shown at the bottom of each tracing. Linear–linear plots of the Fourier power spectra obtained from unfiltered current responses are shown at the right of the corresponding tracing. The orange arrows indicate the line frequency contamination in each preparation.

To further explore the rich oscillatory behavior of isolated MTs, similar experiments were conducted under identical conditions in MT sheets from the same preparation (Fig. 7). The unattached pipette showed a predominantly white noise spectrum with contaminant power line frequency peaks at 50 and 100 Hz (Fig. 7A,D), while the isolated MT-attached spectra displayed colored peaks of relevant frequencies in the oscillatory behavior. We observed clear differences with the MT sheets (*n* = 4), showing fundamental oscillatory frequencies around 38 and 90 Hz (Fig. 7B,D). In all cases, the frequency peaks were richer in the isolated MTs compared to their corresponding MT sheet (Fig. 7A–C, Right). Thus, MTs in the larger surface area of the MT sheet (with a tighter seal) displayed more coherent behavior regarding the observed fundamental frequencies. Thus, an interesting electrical difference between isolated MTs and MT structures became evident, namely, a richer oscillatory behavior of isolated MTs that drastically changed by assembling into flat surfaces showing fewer but stronger oscillatory peaks (Fig. 7).

### Calculation of the power and energy dissipated by an isolated microtubule

The power radiated by the oscillating MT can be obtained as an equivalent wire that allows the flow of an alternating current. Although the wave equation of a sheet of brain MTs requires up to seven fundamental frequencies with their respective weight and phase^18^, in this case, for simplicity we assumed an oscillating structure with a single fundamental frequency *f*, such that *ω* = 2*πf*. The mean value of such alternating current is zero. However, the mean square values (both generated current *I*, and voltage *V*) are not. Thus, the non-zero effective values are calculated, where the effective voltage is ${V}_{eff}=\sqrt{\left(1/2\right){V}_{max}^{2}}=0.707{V}_{max}$, and likewise the effective current is ${I}_{eff}=\sqrt{\left(1/2\right){I}_{max}^{2}}=0.707{I}_{max}$, such that $V_{eff} = ZI_{eff}$, where *Z* is the impedance of the equivalent circuit connecting the MT with the pipette. Under resonance (pure dissipative conditions), *Z* = *R,* and the phase angle between parameters φ = 0 and cos φ = 1. However, normally *Z* > *R*, such that the factor cos *φ* = *R/Z*, relates them.

**Figure 8:**
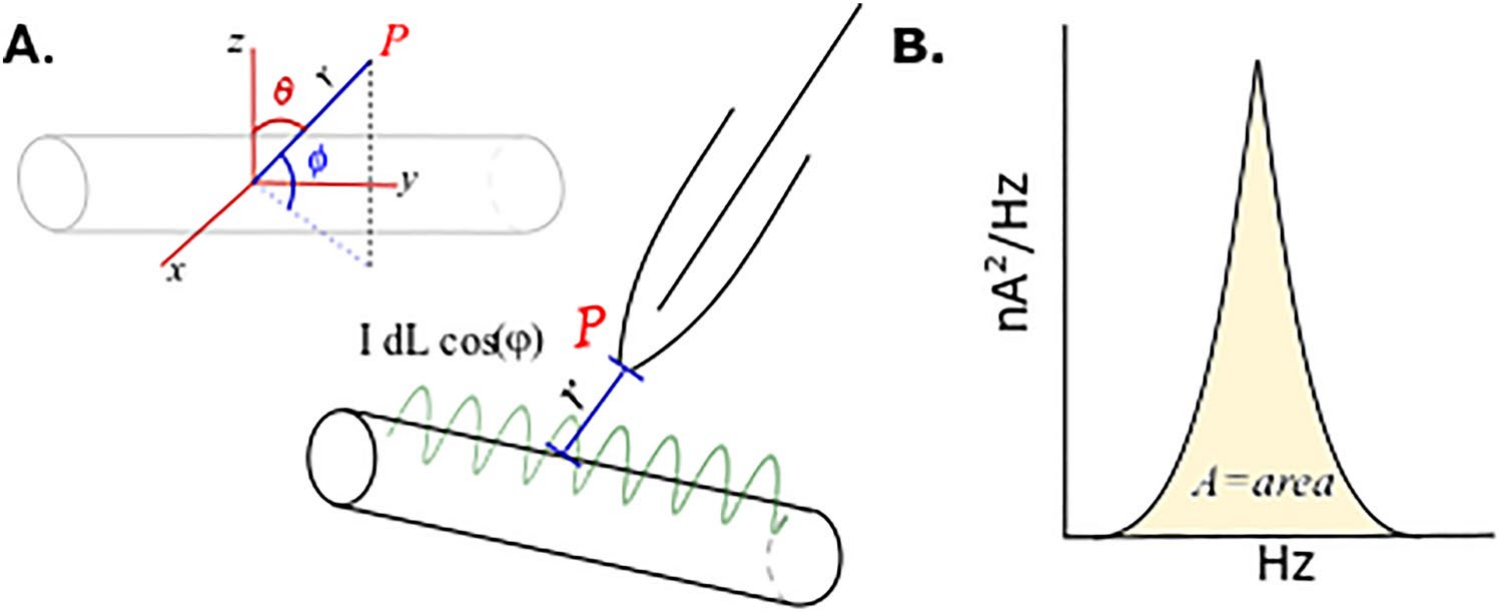
Theoretical model for the calculation of power dissipated by MT. (**A**) Top Left, Electric and magnetic fields sensed by point *P*, at a distance of the MT, represents the collection place at the pipette tip, as indicated in the diagram at bottom right. Calculations to accomplish the magnitude of both fields and the power ***P***= *(E* x ***H)*** (the Poynting theorem) require the integration of the surface integral of the entire length of the MT, named L. (**B**). The experimental value of the peak current generated by the MT in the absence of an electrochemical gradient, as observed by its variance in the Fourier spectrum is integrated to obtain the power, experimentally, as ***P*** = *RI*^2^, where, *R* represents the radiating resistance of the MT.

Although the instantaneous power (***P***) of the MT is zero, its mean value is ${P}_{mean}={V}_{eff}{I}_{eff}cos\varphi$, where cos *φ*, as indicated above, is the power factor, which has to be considered from the intrinsic values of the dissipating (*R*), and energy storing components of the equivalent circuit representing the connection between the pipette and the MT (Fig. 8).

By definition, the instantaneous power of the current element is $P=R{I}^{2}$, where the mean value is ${P}_{mean}=R{I}_{mean}^{2}$, which is equivalent to ${P}_{mean}={V}_{eff}{I}_{eff}cos\varphi$.

To calculate the angle of power radiated by the oscillating MT that is sensed by the pipette, we apply the vectorial analysis of the Poynting theorem (${\varvec{P}}={\varvec{E}}\times {\varvec{H}}$) establishing the connection between the electric and magnetic field intensities generated by the oscillator. If we place a current element of length *dL* from the MT, where *L* is the length of the MT, with the value *IdL*cos *ωt*, (where *ω* = 2π*f*), the field intensities sensed by a point *P* (Fig. 8) at a distance *r* where the pipette tip is located, three field contributions (in spherical coordinates *r*, *θ*, *ϕ*), are sensed, which are perpendicular to each other. The power Poynting vector is then obtained by the cross product (${\varvec{P}}={\varvec{E}}\times {\varvec{H}}$) (to be developed elsewhere). At point *P*, there are two contributions to the electric field, in the *r* direction (***E***_*r*_), and the *θ* direction (***E***_*θ*_), whereas the orthogonal magnetic field is in the *ϕ* direction (***H***_*ϕ*_). Thus, ${{\varvec{P}}}_{{\varvec{\theta}}}=-{{\varvec{E}}}_{{\varvec{r}}}\times {{\varvec{H}}}_{{\varvec{\theta}}}=0$ and ${{\varvec{P}}}_{{\varvec{r}}}={{\varvec{E}}}_{{\varvec{\theta}}}\times {{\varvec{H}}}_{\phi }=0$. The total dissipated power *P*_*T*_ is then obtained from the surface integration of the mean power, such that ${P}_{T}={R}_{r}{I}_{eff}^{2}$, where *R*_*r*_ is the radiation resistance. The importance of these equations is their ability to provide a means to calculate the energy emanated from a single MT that could then be used to compare with the intensity of fields sensed from single axons and cells, and other values obtained by methods such as the EEG of the human scalp.

To apply the theory to our results, we first calculated the area under the curve of the power spectrum around 41.5 Hz (fundamental frequency peak observed in all the experiments without Paclitaxel, Fig. 8), obtaining a variance of 2.65 × 10^–6^ nA^2^. In what follows, the dissipating resistance of the pipette-MT connection used was 10 MΩ, with the caveat that most of it would represent the resistance of the pipette and not the MT resistance.

$${P}_{mean}=R\times {I}_{mean}^{2}={10}^{7}\Omega \times \left(2.65\times {10}^{-6}\times {10}^{-18}{A}^{2}\right)=2.65\times {10}^{-17} \; \text{W}$$

From the consideration above, we know that ${P}_{mean}={V}_{eff}{I}_{eff}cos\varphi$, and we will assume that cos φ = 1. From our experiments, we calculate an *I*_*max*_ of ~ 60 pA, where ${I}_{eff}=0.707{I}_{max}$, such that

$${I}_{eff}=0.707{I}_{max}=0.707\times 60 \times {10}^{-12}A=42.4 \; \text{pA}$$

Replacing, we obtain:

$${V}_{eff}=\frac{{P}_{mean}}{{I}_{eff}cos\varphi }=\frac{2.65\times {10}^{-17} \, \text{W}}{42.4\times {10}^{-12}\, \text{A}}=0.063\times {10}^{-5}\, \text{V}=0.63 \; \upmu\text{V},$$

which is the electrical driving force present in the MT in the absence of an external holding potential, that drives the oscillatory current. By applying the Nernst equation an estimation could be made of the ionic concentrations that generate such intrinsic MT potential (between MT lumen and bathing solution). We obtain:

${V}_{eff}=60mVlog\frac{{C}_{1}}{{C}_{2}}$, where *C*_1_ and *C*_2_ are the MT lumen and bathing solutions, respectively. Thus,

$$\frac{{C}_{1}}{{C}_{2}}={10}^{\left(\frac{{V}_{eff}}{60 \; \text{mV}}\right)}={10}^{\left(\frac{6.3\times {10}^{-7} \; \text{V}}{6\times {10}^{-2} \; \text{V}}\right)}=1.0000242$$

For a 140 mM KCl solution:

$${C}_{1}=1.0000242 \; {C}_{2}=1.0000242\times 140 \; \text{mM}=140.00338 \; \text{mM}$$

Thus $$\Delta =\left({C}_{1}-{C}_{2}\right)=0.00338 \; \text{mM}=3.38 \; \upmu \text{M}$$,

Now, we are able to make an estimation of the intra-microtubular volume, *Vol*_MT_ of an ideal 1 µm length MT, with an internal diameter of 10 nm, such that.

$${Vol}_{MT}=\pi \times {r}^{2}\times l=\pi \times {\left(\frac{{10}^{-8} \, \text{m}}{2}\right)}^{2}\times {10}^{-6} \; \text{m}=7.85\times {10}^{-23} \; { \text{m}}^{3},$$

from which we could infer the minimal charges implicated in the voltage difference. Thus, recalculating with the minimal *ΔC* necessary to generate a voltage in the pipette tip considering *Vol*_*MT*_, we obtain a luminal concentration of cation (K^+^) of 2.65 × 10^–25^ mol, that represents a total charge *Q* = *FC* of 2.56 × 10^–20^ C, where *F* has the usual meaning of 96.500 C/mol.

## Discussion

While the mechanical and biochemical aspects of MT formation and stability have been thoroughly studied, their electrical properties and ability to generate electromagnetic fields have not. Here we demonstrated that isolated MTs generate electrical oscillations, which constitute the basis of their electromagnetic power. Previous studies with Paclitaxel-stabilized brain MTs showed the propagation and amplification of electrical signals but not the presence of electrical oscillations^4,5^. In those studies, MTs amplified the electrical pulse injected at the stimulus and the collection sites, with transfer amplification ratios up to 2.35 under high ionic strength conditions. In retrospect, the absence of oscillations was hypothesized to be caused by the use of Paclitaxel to stabilize and hold the MTs to the pipettes^4,5^. However, electrical oscillations have been observed in MT sheets and bundles that were readily inhibited by the addition of Paclitaxel^7,8^. Thus, herein, we explored Paclitaxel-free, non-stabilized single brain MTs, which instead made it more difficult to find and handle. For comparison, we also studied Paclitaxel-stabilized MTs, without adding the drug to the pipette tip or saline solution during the experiment. We observed that the oscillatory behavior of the isolated MTs was different from that of MT sheets present in the same preparation. In particular, we consistently observed a broader spectrum of fundamental frequencies in the isolated non-stabilized MTs compared to the MT sheets. Paclitaxel-stabilized MTs sustained electrical oscillations with a more restrictive power spectrum than the non-stabilized MTs, showing a single fundamental frequency around 39 Hz, similar to that observed in the more structured MT complexes (i.e., bundles and sheets). This finding raises the hypothesis that assembling MTs into higher structures (e.g., cilia and flagella) may tend to entrain MT oscillations. Different oscillatory modes have been postulated for other MT structures^7,8^.

We present evidence for the intrinsic electrical properties of MTs without external stimulation. At zero mV applied holding potential, under symmetrical ionic conditions (lack of an electrochemical gradient), the oscillatory current generated by an isolated MT in solution was evidenced over the noise of its respective patch pipette alone. The power spectrum revealed the presence of minimal oscillations, which in the absence of holding voltage could only be sustained by the existence of a driving force, likely generated by an electrochemical gradient originated by the geometrical parameters of the MT and the permeable ionic species between the MT lumen and the bath solution. To calculate the power radiated by the oscillating MT and sensed by the pipette, we applied the Poynting theorem, establishing the connection between the oscillator's electric and magnetic field intensities. The power Poynting vector is then obtained by the cross product (***P = E*** x*** H***).

An analysis of the power spectrum at zero mV of an MT in the absence of Paclitaxel, revealed that the area under the curve in the region 43–47 Hz was 2.65 × 10^–6^ nA^2^ from which we calculated a *V*_eff_ at the pipette tip of 63 µV which could be maintained by a K^+^ concentration difference as little as 3.38 µM. Thus, a minimal concentration difference can generate and sustain electrical oscillations in MTs, consistent with a radiating power in the order of 2.65 × 10^–17^ W per MT. Thus, MTs contribute to the electrodynamic properties of the cell in which they are immersed. In comparison, the power dissipated by the neuronal cytoskeleton of the permeabilized brain in the honeybee, as recently measured by local field potentials^17^, was approximately 300 times larger. The electrodynamic properties of the MTs may contribute to the electric fields measured in EEGs and other technical approaches to assess the vector fields generated by the brain's electrical activity. MT electrical oscillations in the neuronal environment may provide a novel means for electrical interactions between different cellular organelles or cytoskeletal structures, such as the actin cytoskeleton^16^. They may explain the existence and propagation of traveling waves recently ascribed to endogenous electric fields^18–22^. Interestingly, Freedman et al.^12^ predicted a current–voltage relationship of an MT, calculated using a resonant circuit model for a cationic conductance in the order of 10 nS, in agreement with our results. Moreover, the conductance was predicted as dependent of the length on the MT. Length and structure of the MTs, including nanopore density could be the reason(s) for the existence of low and high conductance levels.

The MT electrical oscillations are mechanistically consistent with the behavior of an organic electrochemical transistor^23,24^ that supports amplification and self-sustained current (and voltage) oscillations. In this hypothetical mechanism, a gate region of the electrochemical transistor^4,25^, associated with uneven charge distribution on the MT’s surface^26^, would drive the opening of the ion-permeable nanopores that elicit the electrodiffusional circuit. Thus, electrostatically-induced vibrations of adjacent αβ tubulin heterodimers may act as electrical oscillators allowing electrodiffusional ionic transport through the nanopores. The gating mechanism would share similarities with piezoelectric materials to elicit electromechanical transduction such that their mechanical and electrical properties differ upon voltage-induced changes in conformational state^10^. The memristive behavior unmasked by a voltage-dependent capacitance in non-oscillating MT sheets suggested that the electrical response has a complex voltage-dependent nonlinear response. However plausible, this hypothesis and its implications will require further investigation. Nonetheless, in pursuing a better understanding of the molecular nature of the electrical oscillations, we have focused on our prior findings that the oncology drug Paclitaxel (Taxol), an MT stabilizer that traverses the nanopores of the MT wall^26–29^, inhibits the oscillatory phenomenon^4^, but not the ability to amplify electrical signals.

Interestingly, the observed oscillation frequencies in isolated MTs were similar to those previously reported by empirical mode decomposition (EMD) of brain MT sheets, which also resembled the physiological brain electrical signals commonly observed in EEG^18^. Identified human brain waves associated with normal and pathological brain functions include low-frequency delta waves in the range of 0.5–4 Hz, followed by increasing frequency theta waves, 4–8 Hz, alpha 8–13 Hz, beta 13–30, and finally gamma waves beyond 30 Hz, from which lower (~ 30 Hz) and higher (~ 90 Hz) gamma patterns are also recognized^30,31^. The electrical activity of MTs would be an essential contribution to brain activity, and they represent the intrinsic unit of a central brain oscillator recently postulated for the honeybee brain^17^.

The electrical activity of isolated MTs may offer a novel paradigm in neuronal signaling. Tubulin protofilaments, which either curl into MTs or attach laterally to form a 2D-sheets^7,32^ by lateral apposition of protofilaments spontaneously extend to larger flat surfaces generate electrical signals, whose oscillatory frequency and amplitude would likely depend on the specific composition of the nanopores in the MT wall. The present study sheds new light on the structural/functional correlates of MT electrical activity.

## Materials and methods

### Preparation of isolated MTs

For the present study, we used commercial bovine brain tubulin (catalog No T238, Cytoskeleton, Denver, CO, USA). Tubulin was polymerized with a “hybrid” protocol in either the presence or absence of Paclitaxel (Taxol Equivalent, Invitrogen™, P3456), following general guidelines from the Mitchinson Lab’s online protocols (http://mitchison.med.harvard.edu/protocols/). Briefly, all reactions were conducted in BRB80 solution containing 80 mM PIPES (1,4-piperazinediethanesulfonic acid), 1 mM MgCl_2_, 1 mM EGTA, and pH 6.8 with KOH. MT sheets were obtained as previously reported 7.

In the experiments where MTs were polymerized in the presence of Paclitaxel (stock solution, 10 mM), the drug was initially prepared as per the manufacturer's recommendation and diluted to obtain solutions between 2 and 200 mM, which were added sequentially every 5 min to the tubulin suspension as a dilution 1:10 in BRB80, DTT and GTP.

### MT labeling and identification

MTs were immunolabeled with anti-α-tubulin antibody (H-300, sc-5546, Santa Cruz Biotechnology Inc) at 1:200 dilution^6,7^, and FITC-tagged bovine anti-rabbit IgG-R as the secondary antibody (sc-2367, Santa Cruz Biotechnology Inc, CA) used at a 1:200 dilution. Samples were viewed under DIC and fluorescence microscopy with an IX71 Olympus inverted microscope connected to a digital CCD camera C4742-80-12AG (Hamamatsu Photonics KK, Bridgewater, NJ). Images were collected with the IPLab Spectrum (Scanalytics, Viena, VA) acquisition and analysis software running on a Dell-NEC personal computer.

### Electrophysiology and data analysis

The electronic setup consisted of an E-patch amplifier (Elements, Cesena, Italy) directly connected to the MT via a saline-containing patch pipette, as previously reported^7,8,17^. The electrical signals were recorded on a personal computer. Patch pipettes were made from soda lime capillary tubes (Biocap, Buenos Aires, Argentina) with a 1.25 mm internal diameter and a tip diameter of ~ 4 μm^2^ that rendered a tip resistance in the order of 4–15 MΩ in an "intracellular" KCl solution containing, in mM: KCl 140, NaCl 5, EGTA 1.0, and HEPES 10, adjusted to pH 7.18 with KOH. The experiment was initiated by identifying an immunolabeled MT (Fig. 1A) by fluorescence and DIC, approached by the patch pipette, and gently suctioned to the pipette tip (Fig. 1B). No attempt was made to increase tip resistance further. Holding potential was applied from the amplifier. Electrical signals were analyzed with the software suite pCLAMP 10.0 (Molecular Devices, San José, CA, USA). Sigmaplot Version 10.0 (Jandel Scientific, Corte Madera, CA, USA) was used for statistical analysis and graphics.

### Correction of voltage for mean current-to-voltage relationships.

To obtain the *I-V* relationships shown in Fig. 4A, we applied the loose patch correction to the voltage for each experiment considered. Thus, the voltage at the pipette tip (*V*_*p*_) is given by^33^:

$${V}_{p}={V}_{cmd}\left(\frac{{R}_{s}}{{R}_{s}+{R}_{p}}\right)$$

where *R*_*p*_, *R*_*s*_, and *V*_*cmd*_ are the pipette resistance, seal resistance, and command voltages, respectively (see details in^8^).

### Other current analyses

Average currents were obtained by integrating one-second tracings expressed as mean ± SEM (*n*), where (*n*) represented the number of experiments analyzed for a given condition. To calculate the electrical contribution of the isolated MT in the absence of an electrochemical gradient (i.e., symmetrical ionic conditions and zero mV holding potential), we calculated the variance at 0 mV. We randomly selected ten seconds from recordings of different experiments (*n* = 6) obtained either with the pipette in solution before or after attachment to an isolated MT. For all experiments, we measured the standard deviation of the average tracing using Clampfit 10.0, and calculated the variance as the standard deviation squared. The values of each group were averaged and compared using Student's t-test. Statistical difference was considered when p < 0.05. The Fourier transform subroutine of Clampfit 10.0 obtained power spectra of the unfiltered tracings. Poincare diagrams were constructed by the time delay (τ) approach, where the lag time τ was chosen arbitrarily at 2*f*, and *f* was the data acquisition sampling frequency.

### Schematics

The schemes shown in Fig. 1B were produced with the free software Inkscape 0.92.4 (https://www.inkscape.org).

### Solutions and chemicals

Unless otherwise stated, all reagents were obtained from Sigma-Aldrich (St Louis, MI, USA).

## Data Availability

All data are available as reasonable request to the authors.
